# Progranulin is expressed within motor neurons and promotes neuronal cell survival

**DOI:** 10.1186/1471-2202-10-130

**Published:** 2009-10-27

**Authors:** Cara L Ryan, David C Baranowski, Babykumari P Chitramuthu, Suneil Malik, Zhi Li, Mingju Cao, Sandra Minotti, Heather D Durham, Denis G Kay, Christopher A Shaw, Hugh PJ Bennett, Andrew Bateman

**Affiliations:** 1 Endocrine Research Laboratory, Royal Victoria Hospital and Department of Medicine, McGill University Health Centre Research Institute, 687 Pine Avenue West, Montreal, Quebec, H3A 1A1, Canada; 2 Montreal Neurological Institute, McGill University, Montreal, Quebec, H3A 2B4, Canada; 3 University of British Columbia, Departments of Ophthalmology and Visual Sciences, and Experimental Medicine and Graduate Program in Neuroscience, Vancouver, British Columbia, V5Z 1L8, Canada; 4 Neurodyn Inc., Suite 508, NRC-INH, 550 University Avenue, Charlottetown, Prince Edward Island, C1A 4P3, Canada

## Abstract

**Background:**

Progranulin is a secreted high molecular weight growth factor bearing seven and one half copies of the cysteine-rich granulin-epithelin motif. While inappropriate over-expression of the progranulin gene has been associated with many cancers, haploinsufficiency leads to atrophy of the frontotemporal lobes and development of a form of dementia (frontotemporal lobar degeneration with ubiquitin positive inclusions, FTLD-U) associated with the formation of ubiquitinated inclusions. Recent reports indicate that progranulin has neurotrophic effects, which, if confirmed would make progranulin the only neuroprotective growth factor that has been associated genetically with a neurological disease in humans. Preliminary studies indicated high progranulin gene expression in spinal cord motor neurons. However, it is uncertain what the role of Progranulin is in normal or diseased motor neuron function. We have investigated progranulin gene expression and subcellular localization in cultured mouse embryonic motor neurons and examined the effect of progranulin over-expression and knockdown in the NSC-34 immortalized motor neuron cell line upon proliferation and survival.

**Results:**

*In situ *hybridisation and immunohistochemical techniques revealed that the *progranulin *gene is highly expressed by motor neurons within the mouse spinal cord and in primary cultures of dissociated mouse embryonic spinal cord-dorsal root ganglia. Confocal microscopy coupled to immunocytochemistry together with the use of a progranulin-green fluorescent protein fusion construct revealed progranulin to be located within compartments of the secretory pathway including the Golgi apparatus. Stable transfection of the human *progranulin *gene into the NSC-34 motor neuron cell line stimulates the appearance of dendritic structures and provides sufficient trophic stimulus to survive serum deprivation for long periods (up to two months). This is mediated at least in part through an anti-apoptotic mechanism. Control cells, while expressing basal levels of progranulin do not survive in serum free conditions. Knockdown of progranulin expression using shRNA technology further reduced cell survival.

**Conclusion:**

Neurons are among the most long-lived cells in the body and are subject to low levels of toxic challenges throughout life. We have demonstrated that progranulin is abundantly expressed in motor neurons and is cytoprotective over prolonged periods when over-expressed in a neuronal cell line. This work highlights the importance of progranulin as neuroprotective growth factor and may represent a therapeutic target for neurodegenerative diseases including motor neuron disease.

## Background

The granulin-epithelin precursor, progranulin (PGRN) [1], also called proepithelin [2], PC-cell-derived growth factor [3], or acrogranin [4], is a secreted glycoprotein that promotes mitosis, survival, and migration in many cell types [5,6]. Recent work demonstrates that haploinsufficiency of the *PGRN *gene causes a form of frontotemporal lobar degeneration (FTLD) that is associated with the formation of ubiquitinated inclusions (FTLD-U) [7-9]. Several studies have shown that ubiquitinated Tar-DNA Binding Protein 43 (TDP-43) is a component of inclusion bodies in both FTLD-U and Amyotrophic Lateral Sclerosis (ALS) [10-13] although other ubiquitinated proteins are also present in these inclusion bodies in ALS [14]. TDP-43 translocates from the nucleus to the cytoplasm in axotomized motor neurons which is consistent with a role for TDP43 in the normal response of motor neurons to injury [15]. The depletion of PGRN in H4 gliomas results in the activation of caspase-3 and the accumulation of cleaved TDP-43 [16]. This is suggestive of a functional relationship between the loss of PGRN and mobilization of TDP-43, although this conclusion has been challenged by other investigators [17,18]. While PGRN is secreted by many cell types it has been suggested that in neurons its subcellular distribution resembles that of mitochondria or lysosomal-endosomal markers [19].

PGRN is synthesised in neurons in many brain regions including the cerebral cortex, in the Purkinje cells of the cerebellum, and in the hippocampus [20]. In addition, it is widely distributed in the developing central nervous system and the dorsal root and sympathetic ganglia within the peripheral nervous system [21]. The roles of PGRN in normal neuronal function and development, in either the central or peripheral nervous systems are poorly understood. It is known, however, that PGRN contributes to normal brain development since it regulates the male-specific differentiation of the neonatal hypothalamus [22,23]. Moreover, in culture, PGRN stimulates the proliferation of PC12 cells [20], as well as the estrogen-dependent growth of hippocampal neurospheres [24] and may be neurotrophic for cortical and motor neurons [25].

The signalling pathways associated with PGRN in neurons are unknown, but in non-neuronal cell types it activates growth factor-related signal transduction pathways including the phosphorylation of shc, p44/42 mitogen-activated protein kinase, phosphatidylinositol 3-kinase, protein kinase B/AKT, and the p70^S6 ^kinase [26-28], and, by so doing, contributes to carcinogenesis in numerous tumour types [29-39]. PGRN is involved in wound repair and inflammation [40-43], and plays an important role in early embryonic development [44-46]. The ability of PGRN to regulate critical proliferative, survival and motility signals in a diverse range of non-neuronal cell types suggests that it may support similar functions in nerve cells.

*In situ *hybridization experiments (see below) revealed that motor neurons express a high level of PGRN, suggesting a significant role for PGRN in the biology of the motor neuron. The significance of PGRN in healthy and diseased motor neurons is, however, unclear. PGRN expression was markedly up-regulated in spinal cord tissue from patients who had ALS [47]. While this was probably due to gliosis [47], increased immunoreactive staining for PGRN has been reported both in motor neurons and glial cells in spinal cord and brainstem tissue sections derived from ALS patients relative to controls [48]. Furthermore, PGRN expression is increased in the lumbar cord motor neurons from murine models of familial ALS that is caused by mutations in *superoxide dismutase 1 *(*SOD1*) [49,50]. *PGRN *was one of only 21 genes with dysregulated motor neuron expression in *SOD1*^*G37R *^and *SOD1*^*G85R *^mice at the onset of the disease, as judged by weight loss but before the detection of overt neurological symptoms [49]. The *PGRN *gene was also up-regulated in SOD^G93A ^mice, but late in the disease progression [50]. In contrast, a decrease in PGRN expression was noted in the NSC-34 neuronal cell line engineered to express SOD^G93A ^[51]. It has been suggested that the *PGRN *gene may be a modulator of disease progression in ALS since a correlation between genetic variations in *PGRN *and the age of onset or survival of patients with ALS has been reported [52]. This observation was not, however, reproduced by other investigators [53-55]. In some patients FTLD may be accompanied by motor neuron disease [56,57], but mutations in *PGRN *are infrequent in patients with ALS-FTLD defects [54,58-60], and for those that have been reported it is uncertain whether or not they are pathogenic [53,61].

Growth factors, in particular vascular endothelial growth factor [62] and insulin-like growth factor-1 [63], have shown promise as neuroprotective agents in murine models of familial ALS and if PGRN is neurotrophic for motor neurons [25] it could also have therapeutic potential in ALS. Given the uncertainty of whether or not PGRN contributes to motor neuron function and survival, we sought first to characterize the expression of PGRN in the healthy brain, spinal cord and dorsal root ganglia, and then to investigate its possible biological activities in an immortalized motor neuron cell model.

## Results

### PGRN is expressed within multiple neuronal cell populations of the mouse brain and spinal cord

**Figure 1:**
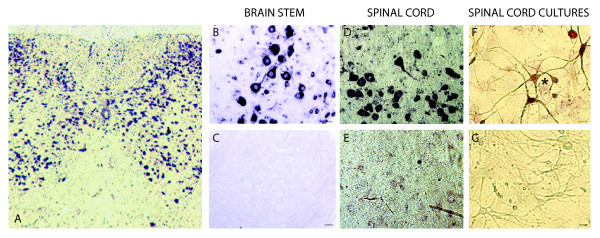
**PGRN is expressed by neurons of both central and peripheral nervous systems, *in vivo *and *in vitro *in the mouse**. Gene expression pattern of murine PGRN in brain (B, C); cervical spinal cord (A, D, E) and primary cultures of dissociated spinal cord-DRG (F, G). *In situ *hybridization, to detect PGRN mRNA in saggital section of pontine grey matter (A) and cross-section of cervical spinal cord (D, E). The majority of neurons throughout the grey matter of the spinal cord express PGRN as well as ependymal cells and possibly microglial cells (A). Note in particular the robust expression of PGRN mRNA in large motor neurons in panels B, D. Panels C and E illustrate the hybridization signal observed with the sense control applied to serial sections to those shown in panels B and D, respectively. (F) Motor neurons (asterisk) as well as other neuronal subtypes in dissociated spinal cord-DRG cultures, express PGRN; (G) equivalent sense control. Scale bar (panels B-G) represents 20 μm. Original magnification of panel A was 10×

The expression pattern of PGRN has been described in the adult brain [21], but not in the remainder of the nervous system. We investigated the expression of PGRN mRNA and protein in normal mouse brain and spinal cord. *In situ *hybridization (ISH) was performed on para-saggital sections of brain, trans-sections of cervical spinal cord, and primary cultures of dissociated spinal cord-dorsal root ganglia (DRGs) (Figure 1). PGRN mRNA was detected within numerous neural cell types within the grey matter of the spinal cord (Figure 1A). Motor neurons in brain (pontine grey matter, Figure 1B) and ventral horn of the cervical cord (Figure 1D) (identification based on a distinctive cell body size (>20 um) [64]) robustly expressed PGRN mRNA. Motor neurons were routinely the first cell population to become visible during chromagen development of the ISH signal. The expression of PGRN mRNA was investigated in primary cultures of murine spinal cord-DRG. PGRN was expressed within motor neurons, as well as other neuronal cells (Figure 1F).

**Figure 2:**
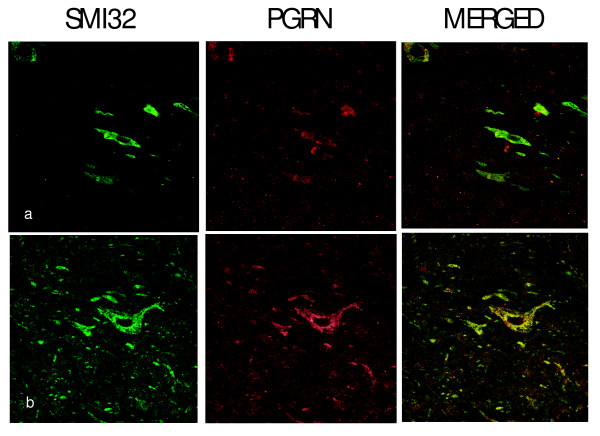
**PGRN is localized within motor neurons of the mouse lumbar spinal cord**. PGRN is localized within motor neurons of the mouse spinal cord. Labelling of paraffin-fixed cross-sections of murine spinal cord with SMI32 marker against hypo-phosphorylated neurofilaments (left panel), which is a marker for motor neurons, and anti-PGRN (middle panel). Merged channels are shown in the right panel. (a) at original magnification (40×), (b) at magnification (63×).

**Figure 3:**
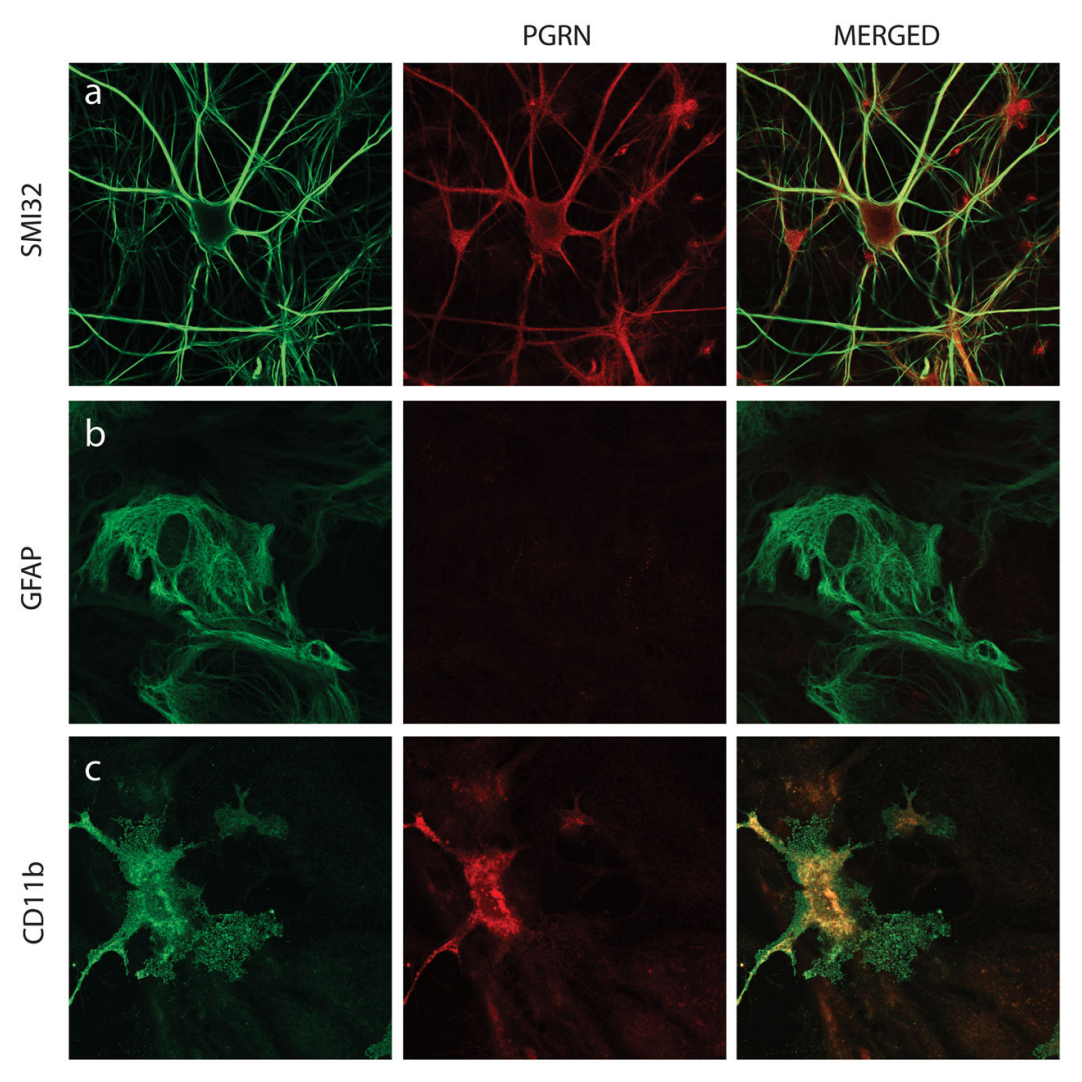
**PGRN expression within the dissociated spinal cord-DRG cultures**. Confocal Images taken of dissociated spinal cord cultures. PGRN (red) is very clearly expressed within motor neurons (a), labelled with SMI32 (green). PGRN is also expressed by microglia, as demonstrated by colocalization between PGRN and CD11b (c). Astrocytes, however, do not express PGRN, as demonstrated in (b).

Expression of PGRN at the protein level was demonstrated by the presence of PGRN-immunoreactive protein within motor neurons in cross-sections of the lumbar spinal cord. Figure 2 shows co-labelling of large neurons in the ventral cord immunoreactive with both anti-PGRN and the neurofilament marker SMI32, which strongly labels motor neurons [64]. SM132 positive signals were found to predominantly coincide with PGRN immunoreactivity. The expression of PGRN protein was also prominent in motor neurons labelled with SMI-32 in primary motor neurons from spinal cord-DRG cultures (Figure 3a) and was also present in CD11b positive microglia in these cultures (Figure 3c). PGRN did not co-express with an astrocyte marker (glial fibrillary acidic protein, GFAP) (Figure 3b). This was not unexpected since these cells are of different lineages. Microglia are derived from hematopoietic stem cells, while astrocytes have oligodendrocyte precursor cells.

### Subcellular localization of PGRN within primary spinal cord motor neurons and NSC-34 immortalized motor neuron cells

**Figure 4:**
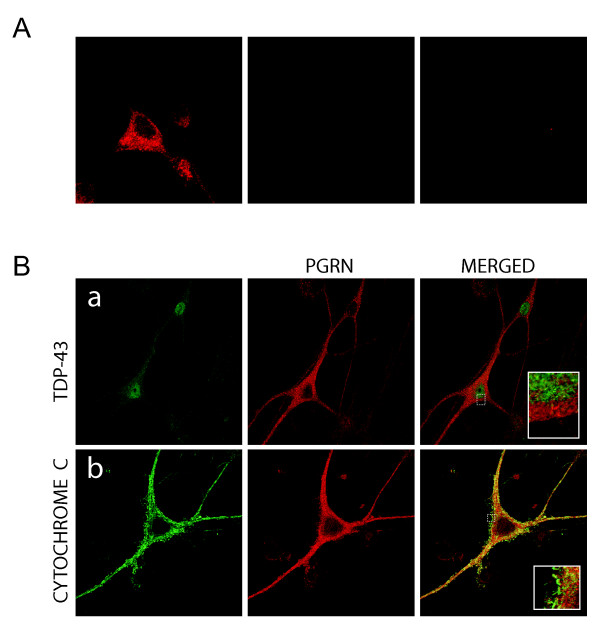
**PGRN within motor neurons in primary cultures does not colocalize with the nucleus or mitochondria**. **(A) **Motor neuron labeled with antibody to mouse PGRN (left hand image) is attenuated by antigen-competition with 300 ng recombinant mouse PGRN (middle and right hand images). When anti-PRGN was pre-absorbed with 400 ng of mouse recombinant PGRN, no signal was observed in the primary motor neurons (not shown). Shown are confocal images taken at 100×. **(B) **PGRN is not distributed in nuclei or mitochondria, organelles that are not part of the secretory pathway. Immunolabelling of motor neurons in dissociated spinal cord-DRG cultures with anti-TDP-43 (a) and anti-cytochrome C (b) and anti-PGRN (middle column). Merged images (right column) show no colocalization of TDP-43 or cytochrome C with endogenous mouse PGRN. Confocal images were captured at 63× magnification, hatched boxes represent 3-5× zoom.

**Figure 5:**
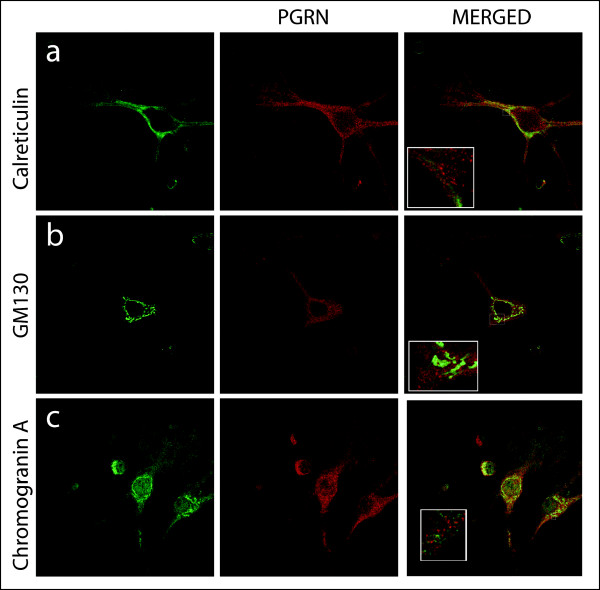
**Subcellular localization of PGRN within motor neurons in primary cultures relative to markers for the ER, Golgi apparatus and chromogranin-A containing vesicles**. Immunolabelling of motor neurons in dissociated spinal cord-DRG cultures with anti-Calreticulin (a), anti-GM130 (b), Chromogranin A (c) and anti-PGRN (middle column). Confocal images were captured at 63× magnification, hatched boxes represent 3-5× zoom.

**Figure 6:**
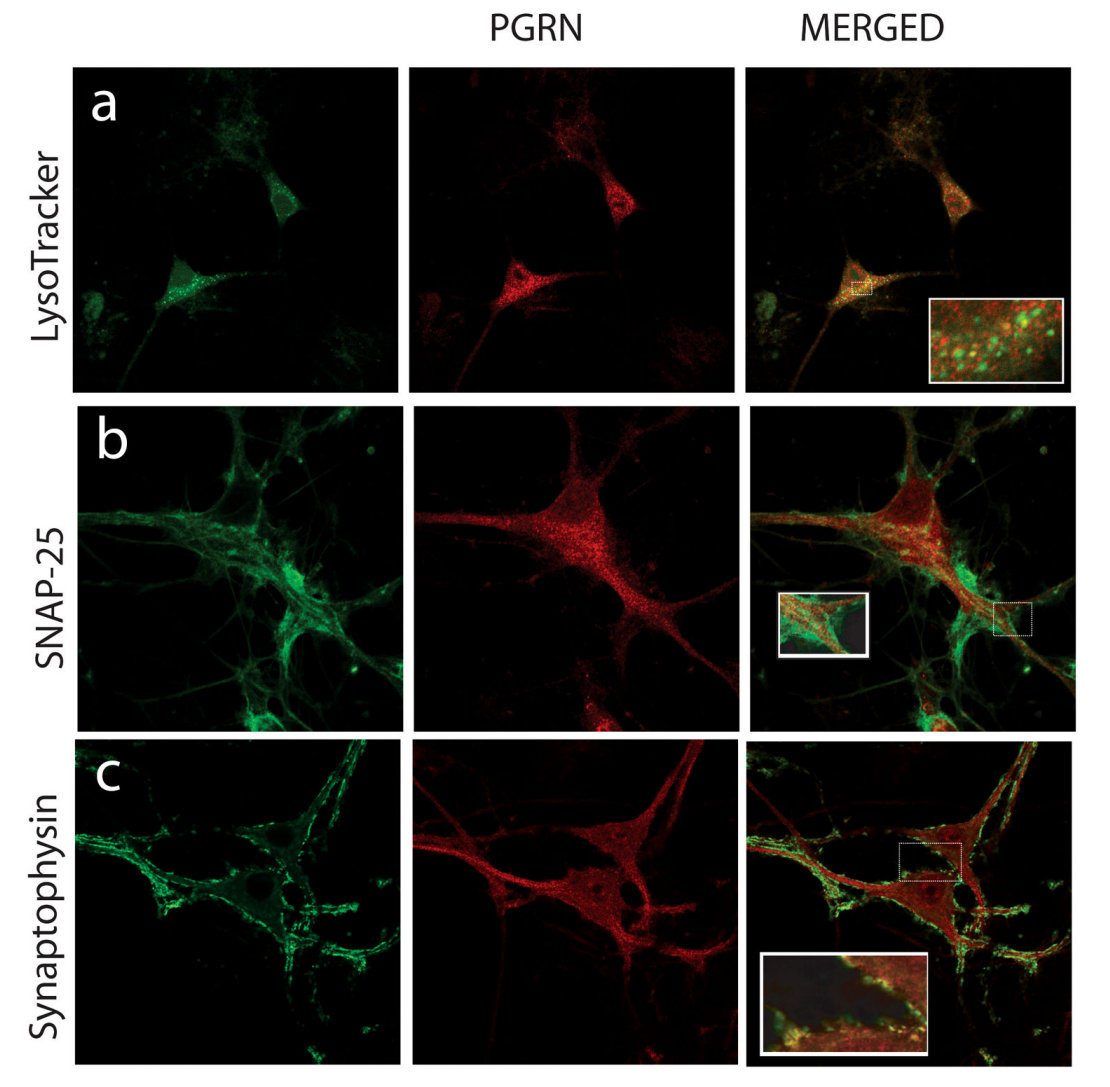
**Subcellular localization of PGRN within motor neurons in primary cultures relative to a marker for the lysosomal compartment markers for neurotransmitter vesicle trafficking and release (SNAP-25 and synaptophysin)**. Immunolabelling of motor neurons in dissociated spinal cord-DRG cultures with LysoTracker™ (a), SNAP-25 (b), Synaptophysin (c), and anti-PGRN (middle column). Confocal images were captured at 63× magnification, hatched boxes represent 3-5× zoom.

Confocal immunofluorescence microscopy was used to investigate the subcellular distribution of PGRN within primary motor neurons. Specificity of the immunoreactivity was validated by antigen competition using murine PGRN (Figure 4A). PGRN is primarily found in the cell body with a punctate distribution within the cytoplasm. However, PGRN expression was also prominent within the axons of neurons. PGRN was not observed within the nucleus as defined by TDP43 immunofluorescence, nor was it present within mitochondria using Cytochrome C immunofluorescence as the mitochondrial marker (Figure 4B-a,b). Interestingly, PGRN immunostaining did not colocalize with calreticulin, a calcium-binding chaperone that facilitates transit of correctly folded proteins and is a marker for the endoplasmic reticulum (ER) (Figure 5a). PGRN expression was also apparently absent from the trans-Golgi network (TGN) as indicated by the marker GM130 (Figure 5b). This finding was surprising, since the biosynthetic precursor to PGRN carries a signal peptide, the structural cue for passage through the secretory pathway. PGRN did not colocalize with another secreted protein, chromogranin A, (Figure 5c); however, the size of PGRN vesicles appeared to be of a similar dimension to that of the chromogranin A-containing vesicles. PGRN did appear to have limited colocalization with synaptophysin (Figure 6c), which is a protein of the synaptic vesicle exocytosis pathway although at the resolution available we cannot exclude the possibility that this is due to a non-specific overlay effect. Few lysosomes, if any were positive for PGRN (Figure 6a). Antibody to PGRN did not colocalize with SNAP-25, a marker for neurotransmitter vesicle docking and release sites (Figure 6b).

### Subcellular localization of PGRN-Enhanced Green Fluorescent Protein in NSC 34 cells

**Figure 7:**
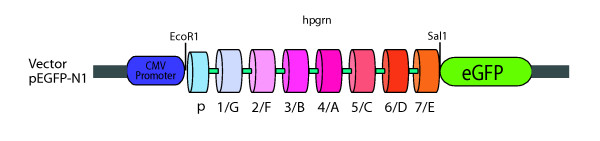
**pEGFP-N1-hPGRN cloning construct**. The human PGRN cDNA (hpgrn) was cloned into the pEGFP-N1 plasmid using EcoR1 and Sal1 restriction enzyme digestion sites. The eGFP molecule is fused to the C-terminus of the PGRN protein, and therefore does not affect its N-terminal signal sequence that is required for entry into the endoplasmic reticulum. Individual 12 cysteine granulin modules are designated 1 through to 7 and A through to G. P is paragranulin, a half-granulin module bearing six cysteine residues.

**Figure 8:**
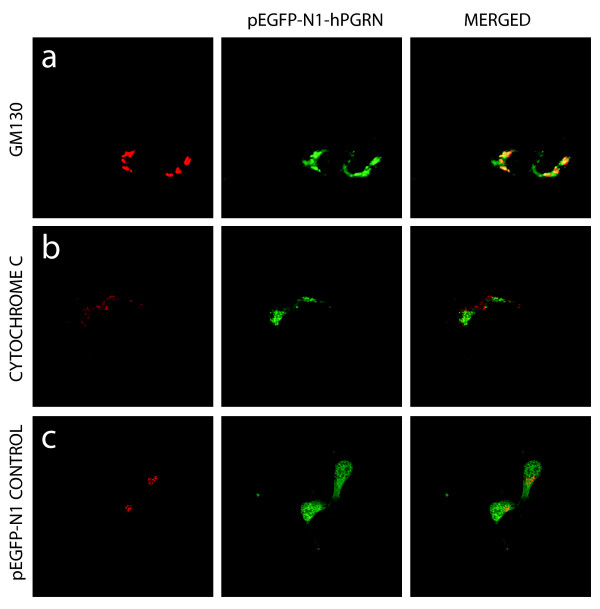
**Transfection of pEGFP-N1-hPGRN plasmid shows granular morphology and particular PGRN localization in Golgi**. The overlap seen with PGRN and the Golgi marker GM130 (a) is very prominent, while there is no overlap between mitochondria (b) and PGRN. Panel (c) reveals the pEGFP-N1 vector only transfection, having primarily nuclear GFP signal, and a very different subcellular distribution of PGRN, which appears distinctly granular in panels (a and b). Confocal images were taken at 63×.

Further studies confirmed that the subcellular distribution of PGRN in NSC-34 cells was comparable to that of the primary motor neurons (data not shown), including the labelling of NSC-34 cells with SMI-32 which is consistent with motor neuron-like properties. Given the disparity between the apparent absence of PGRN immunoreactivity in the ER and Golgi apparatus (Figure 5) but the presence in PGRN of the structural hallmarks for a protein that should enter the ER and Golgi apparatus including a signal peptide, glycosylation and disulfide bridging it was essential to re-evaluate the localization of PGRN using an independent technique. Lacking an extensive selection of commercial anti-PGRN antibodies validated for use in immuno-staining available at the time, we developed a green fluorescent protein (GFP)-tagged PGRN construct to investigate PGRN subcellular localization further. This construct, consisting of an enhanced GFP (eGFP) tag fused to the carboxyl-terminus of the human PGRN gene (named pEGFP-N1-hPGRN), was transiently transfected into NSC-34 cells (Figure 7). 48 hours after transfection, confocal microscopy of the cells demonstrated successful transfection, and a granule-like appearance for the Green Fluorescent Protein signal that was very different from that of control empty eGFP vector-transfected cells in which eGFP vector expression was more nuclear (Figure 8c). The pEGFP-N1-hPGRN expressing cells demonstrated clear colocalization of EGFP-PGRN within the TGN (Figure 8a). Furthermore, PGRN distribution was distinct from that of the mitochondria (Figure 8b). The presence of the eGFP-tagged PGRN in the Golgi apparatus is not secondary to PGRN over-expression since when we over-expressed untagged PGRN in NSC34 cells, as with the primary motor neurons (Figure 5), no untagged-PGRN immunoreactivity was detected in the Golgi apparatus (data not shown).

### Over-expression of PGRN in NSC-34 cells promotes a neuron-like morphology

**Figure 9:**
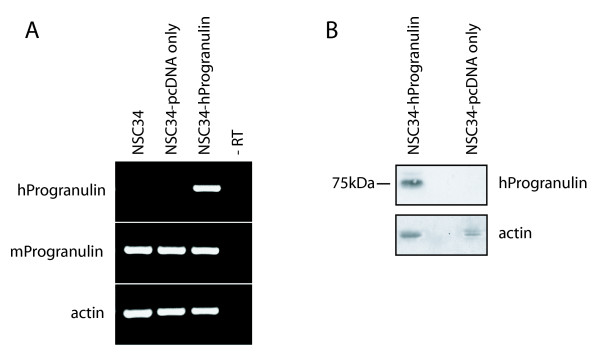
**Validation of Stable Transfectants**. The motor neuron-neuroblastoma hybrid cell line, NSC-34, was transfected with empty pcDNA vector or pcDNA-hPGRN and selected for drug resistance over a three-week period. **(A) **Species-specific PGRN primers were used to confirm the stable integration of hPGRN. **(B) **Western Blot analysis confirmed presence of hPGRN in stable transfectants and not in the vector only control cells.

**Figure 10:**
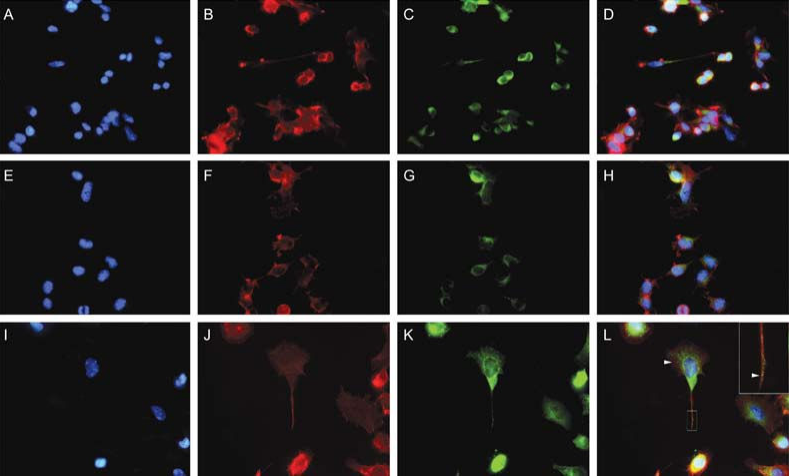
**PGRN over-expression in NSC-34 cells promotes a neuronal morphology**. Untransfected NSC-34 cells (A-D), transfected with pcDNA3 vector only (E-H) or transfected with pcDNA3-hPGRN (I-L). Micrographs showing the distribution of DAPI (A, E, I), F-actin (B, F, J), hPGRN (C, G, K) and merged images (D, H, L). PGRN over-expression promotes more extensive cytoskeletal extensions (hatched box) and is localized within presumptive secretory granules (arrowheads).

The cell line NSC-34 was established by fusing embryonic spinal cord cells with neuroblastoma cells and in the differentiated state are reported to exhibit motor neuronal properties [65,66]. They are frequently used to investigate neuroprotective processes and to model motor neuron degeneration *in vitro *[51,67-72]. In culture most NSC-34 cells exhibit a rounded and undifferentiated morphology; however, serum-deprivation stimulates many of the cells to undergo differentiation and to extend neurite-like projections [66,73]. Serum-deprivation is also associated with significant apoptosis of the non-differentiated cells [73]. NSC-34 cells that express human PGRN were established by transfection and selected for stable incorporation of the human *PGRN *gene. Previous work had demonstrated that human PGRN is mitogenic for murine cells [28]. Untransfected NSC-34 cells, NSC-34 transfected with empty PCDNA3 vector (NSC-34/vector), and NSC-34 cells that were transfected with human *PGRN *cDNA subcloned into pcDNA3 (NSC-34/PGRN) continued to express equivalent levels of murine PGRN mRNA, while only the NSC-34/PGRN cells express the human PGRN mRNA (Figure 9A). Over-expression of PGRN protein was confirmed by Western blot analysis (Figure 9B). In NSC-34/PGRN cells, PGRN punctate immunoreactivity was found throughout the cell body and within the projections (Figure 10). Over-expression of PGRN was associated with morphological changes, such as flattening of cell shape and more pronounced neurite-like extensions (Figure 10).

### PGRN over-expression promotes cell survival

**Figure 11:**
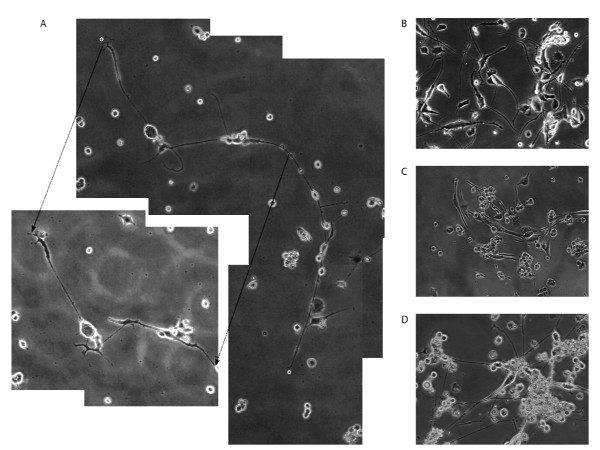
**PGRN is a sufficient trophic stimulus to maintain prolonged survival of NSC-34 cells in serum-free medium**. NSC-34 cells stably transfected with pcDNA3/PGRN and grown in serum-free medium **(A) **for 20 days in six well plates, the cut-out box illustrating the same cells photographed 3 hr later showing continued active extension and retraction of processes; **(B) **for 51 days, and **(C, D) **for 67 days in serum free medium. All NSC-34/vector control cells died before day 20 (not shown). Images were taken at an original magnification of 15×.

To investigate the longevity of NSC-34/PGRN cells, 2 independent cultures were propagated for 2 months in the absence of serum. Within 20 days no NSC-34/vector cells survived (not shown), however many NSC-34/PGRN cells remained alive, retaining a mixed morphology of undifferentiated cells and cells with complex neurite-like projections [Figure 11A, B, C]. Cells with neuron-like morphology remained viable at least for 2 months in the absence of serum [Figure 11C, D]. At day 20, when compared over short intervals (3 hrs) the neuron-like NSC-34/PGRN cells were changing morphologically, continuing to elaborate and elongate neuritic processes [Figure 11A].

**Figure 12:**
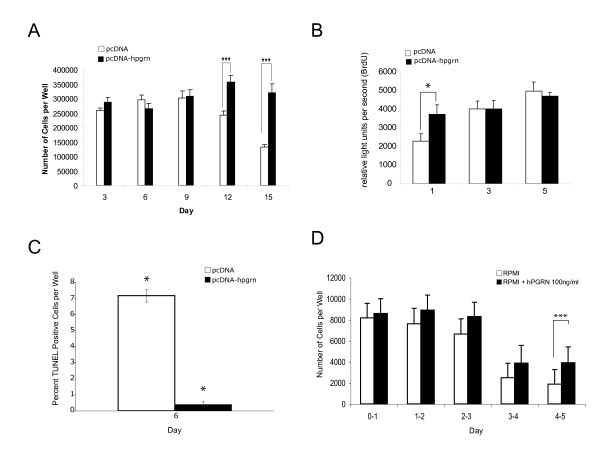
**PGRN over-expression prevents apoptosis of NSC-34 cells induced by serum deprivation and exogenous PGRN increases cell survival**. Stable vector only transfectants (pcDNA, open bars) and cells stably over-expressing hPGRNs (pcDNA-*hPGRN*; black bars) were cultured in serum-free RPMI medium in six well plates. **(A) **Number of cells per well were determined at three-day intervals for fifteen days. NSC-34 cells that over-expressed hPGRN demonstrated increased survival as compared to controls (N = 2 6 fields 10× magnification/each condition, Asterisks denote P < 0.005). **(B) **Cell proliferation assay based on 18 hr BrdU incorporation following 1, 3 and 5 days culture in serum-free medium in 96 well plates. Over-expression of hPGRN during serum deprivation did not significantly increase cell proliferation rates (10 replicates per measurement P > 0.1). **(C) **Apoptosis assay based on the TUNEL- labelling method following 6 days in serum-free medium. Over-expression of hPGRN during serum deprivation protected against apoptosis (N = 2 6 fields 10× magnification per condition. Asterisks denote P < 0.0001). **(D) **Addition of exogenous PGRN also dramatically increased NSC-34 survival (P < 0.0001).

Between 12 and 15 days following the removal of serum, cell number significantly decreased in NSC-34/vector cultures, while cultures of NSC-34/PGRN cells showed no such change (Figure 12A). The absence of extensive proliferation in NSC34-PGRN cells cultured in serum free medium was further confirmed using a BrdU incorporation assay. No significant difference in BrdU incorporation between the NSC-34/vector and NSC-34/PGRN cells was observed on days 3 and 5. (Figure 12B). There was, however, a significant increase in TUNEL-positive (i.e. apoptosing) cells in the NSC-34/vector cultures versus the NSC-34/PGRN cultures (Figure 12C). The addition of exogenous recombinant human PGRN to the medium also resulted in an enhanced cell survival that became evident at day 3 of the incubation (Figure 12D).

**Figure 13:**
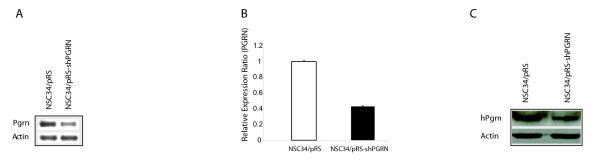
**Short hairpin RNA silencing of PGRN in NSC 34**. **(A) **RT-PCR of PGRN and actin in NSC34 cells stably -transfected with shRNA. **(B) **Relative expression ratio of quantitative RT-PCR showing the expression of PGRN mRNA in NSC34/pRS plasmid and NSC34/shPGRN. **(C) **Western blot analysis of PGRN and actin in NSC34 cells with stably-transfected with shRNA.

**Figure 14:**
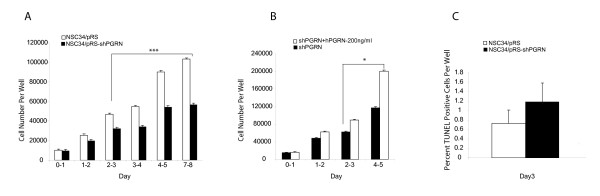
**PGRN knockdown reduces cell proliferation and exogenous PGRN rescues cell proliferation**. **(A) **AlamarBlue cell proliferation assay allowing the quantitative measurement of cell proliferation from day 0 to day7 in the presence of serum in 96 well plates. PGRN knockdown significantly reduced the cell proliferation rate from day 2-3 to day 7-8 (10 replicates per determination, p > 0.001). **(B) **Addition of exogenous PGRN rescued NSC-34 cell proliferation from day 2-3 to 4-5 (representative experiment, 10 replicated per determination, P < 0.05). **(C) **Apoptosis assay based on TUNEL labelling method following 3 days in the presence of serum. PGRN silencing had no significant effect on apoptosis (N = 2, 6 fields 10× magnification per determination).

The stable expression of an shPGRN construct reduced PGRN mRNA levels by approximately 50% (Figure 13A, B). Consistent with the qRT-PCR data, significant decreases in the level of PGRN protein was observed in the shRNA expressing cells as confirmed by Western blot analysis (Figure 13C). When the shPGRN cells were cultured in serum-containing medium, cell proliferation was significantly reduced relative to the vector control cells (Figure 14A) but there was no statistical change in the proportion of apoptotic cells (Figure 14B). The reduced proliferation of shPGRN was reversed by the addition of exogenous human PGRN to the culture medium (Figure 14C).

## Discussion

Mutations of the *PGRN *gene cause frontotemporal lobar degeneration accompanied by the appearance of ubiquitinated-inclusion bodies [7,8]. The formation of ubiquitinated inclusions occurs in other neurodegenerative diseases, in particular in ALS [12]. While mutations of *PGRN *do not appear to cause ALS [53,54], recent work suggests that PGRN is neurotrophic for spinal cord motor neurons [25]. Here we confirmed that PGRN mRNA and protein is expressed in mouse spinal motor neurons, both *in situ *and in primary culture (Figures 1, 2, 3). Other spinal cord neurons, and spinal cord microglia also express PGRN. The expression in microglia (Figure 3), which also has been seen in the brains of Alzheimer's patients [8], is of interest, given that microgliosis accompanies motor neuron degeneration. Recent evidence demonstrates that in peripheral inflammation, PGRN inhibits the activity of the pro-inflammatory cytokine tumor necrosis factor-alpha [41,43], and it is possible that PGRN may also regulate inflammatory processes in the spinal cord.

Cells of different origins handle PGRN in different ways. Whereas epithelial cells appear to secrete PGRN constitutively, innate immune cells including neutrophils process this growth factor to yield 6 kDa granulin peptides that are stored within granules [74]. Similarly, PGRN is stored within the acrosomes of guinea pig spermatozoa [4], which are vesicular storage structures. In addition, yeast-two hybrid studies have identified PGRN as a potential partner for nuclear proteins such as cyclin-T [75], adding a further level of complexity to the localization and roles of PGRN.

The subcellular localization and fate of PGRN in neurons has not been previously defined. PGRN appears to be a strong candidate for entry into the ER/Golgi pathways since the PGRN gene encodes a signal sequence for co-translational entry into the ER, it is rich in disulfide bridges and N-glycosylation sites both of which form during transit through the ER and Golgi compartments. However, it has been suggested that in nerve cells PGRN may localize with either mitochondria or endosomal-lysosomal-like structures [19], which, if correct has profound implications with respect to the mechanism of action of PGRN. It was therefore important to clarify the sub-cellular compartmentalization of PGRN in nerve cells.

Punctate PGRN immunofluorescence was observed within the cell bodies and in the axons of primary motor neurons in culture, but not in their nuclei (Figures 4, 5, 6). We were unable to detect any co-localization of PGRN with mitochondria in primary motor neurons in culture (Figure 4B, b). However, we were also unable to detect immunoreactive PGRN in the ER or Golgi apparatus (Figure 5a, b). The absence of PGRN immunoreactivity in the ER/Golgi system despite the structural arguments that suggests it would be likely to enter the secretory pathway, may reflect a genuine dissociation of localization. Given the structural features of mature PGRN this may also be a misleading result due, for example, to low PGRN concentrations in the ER/Golgi resulting from rapid transfer through the secretory pathway. Alternatively the PGRN antibody may not react with immature protein as it transits through the ER/Golgi processing pathways. The monoclonal antibody was raised against recombinant mouse PGRN. It is not known whether it would detect an immature form of the protein, namely the conformation of PGRN found in the ER/Golgi compartment before N-glycosylation and formation of disulfide bridges have been completed. To avoid some of these possible confounding issues we employed an alternate strategy that was independent of immunolocalization to examine the localization of PGRN within neuronal cells. NSC-34 motor neuron-like cells were transfected with a PGRN-eGFP fusion protein (Figures 7, 8) or with an eGFP control alone. The eGFP control exhibited green immunofluorescence that concentrated mostly in the nucleus. In contrast PGRN-eGFP exhibited a punctuate fluorescence in the cell body and axons, but not the nucleus, in a pattern that was also observed for intrinsic PGRN. The PGRN-eGFP fusion protein co-localized with GM130, a marker for the trans-Golgi apparatus, but not with a mitochondrial marker (Figure 8). We conclude, therefore that neuronal PGRN enters the ER/Golgi secretory pathway.

There are three major exit pathways from the Golgi apparatus; namely, to the lysosome, to the regulated secretory pathway, which is characterized by dense core secretory granules often containing neuropeptides, or to the constitutive secretory pathway. We detected only limited colocalization of PGRN with lysosomes (Figure 6a), suggesting that this is at most only a minor destination for PGRN. The vesicle-like PGRN structures in the motor neurons did not colocalize with chromogranin A (Figure 5c), indicating that PGRN does not enter the regulated secretory pathway. There was, however, evidence of some co-localization between PGRN and synaptophysin (Figure 6c), although further investigation using higher resolution techniques are necessary to confirm this. PGRN is unlikely to be secreted primarily from synaptic junctions since it did not co-localize with the SNAP-25 marker for neurotransmitter vesicle docking and release sites (Figure 6b). The use of the NSC-34 PGRN-eGFP system in conjunction with confocal microscopy of primary motor neuron cultures may be useful in defining some aspects of PGRN secretion by neurons.

We used the NSC-34 cell line to investigate the effects of PGRN upon cell growth and survival. Using species-specific reverse-transcription PCR, the expression of human PGRN was confirmed in the NSC-34/PGRN cells, and there was no compensatory alteration in the expression of the murine PGRN mRNA (Figure 9A). PGRN elicited a change in the appearance of the NSC-34 cells, causing a more flattened cell shape and more prominent neuritic extensions (Figures 10, 11). Serum deprivation was employed as an apoptotic challenge. Upon more prolonged incubation in serum-free medium the number of NSC-34/vector cells declined, becoming statistically significantly different from NSC-34/PGRN between days 12 and 15 (Figure 12A). This was due to reduction in apoptosis in NSC-34/PGRN cells since the number of TUNEL-positive cells was significantly lower in cultures of NSC-34/PGRN cells compared to cultures of NSC-34/vector cells (Figure 12C). The overall cell number did not significantly increase in NSC34 cell deprived of serum and, except at the earliest time points, the percentage of BrdU positive cells was not significantly changed (Figure 12B), suggesting that PGRN is cytoprotective rather than proliferative for NSC-34 cells in the absence of serum. The ability of exogenous PGRN to increase the survival of NSC-34 cells was confirmed by incubating the wild type NSC-34 cells with purified PGRN in serum free medium (Figure 12D). In other non-neuronal cells, such as dermal fibroblasts, PGRN is strongly protective against acidosis [76], suggesting that it may play a widespread role in protecting cells against metabolic shocks in their microenvironment.

In order to assess the effect of reduced endogenous PGRN expression upon proliferation, NSC-34 cells were stably transfected with shRNA for PGRN and validated in terms of RNA and protein expression (Figure 13A, B, C). In the presence of 10% serum, the NSC-34 cells actively proliferate. Under these conditions the reduction of PGRN mRNA expression using shRNA silencing decreased cell proliferation by about 50% (Figure 14A), but had no significant effect on apoptosis (Figure 14C). PGRN added back to the culture medium prevented the inhibition of proliferation brought about by PGRN shRNA (Figure 14B). This confirms the specificity of PGRN shRNA and suggests that PGRN acts primarily through an extracellular mechanism. Therefore, PGRN supports both cell survival (Figure 12) and proliferation of NCS-34 cells (Figure 14) and these activities can be functionally separated depending on growth conditions.

The enhanced synthesis of PGRN provided prolonged trophic support for NSC-34 cells in the absence of serum for periods of at least 60 days (Figure 11D). The extended serum-free cultures continued to show a mixed population of rounded and more-differentiated cells, however neuron-like cells were maintained throughout. In these cultures the projections became highly elongated, and were dynamic structures that displayed structural rearrangement over a period of a few hours (Figure 11A). PGRN may promote neurite extension in cortical neurons [25], and in short-term NSC34-PGRN cultures (Figure 10), however we cannot exclude the possibility that the extended length of projections in the long-term serum-deprived NSC-34/PGRN cultures may be due to improved overall cellular health rather than the direct stimulation of neurite outgrowth.

## Conclusion

Primary motor neuron cultures and the NSC-34 cell line provide useful models in which to investigate the cell biology, function and mode of action of PGRN in neurons. We have demonstrated that PGRN is highly expressed in normal spinal cord neurons, and that it enters the ER/Golgi secretory pathway. Over-expression of PGRN in NSC-34 cells was able to substitute for serum as a trophic support for periods of up to 2 months. Purified PGRN added to the medium of serum-deprived NSC34 cells also decreased the rate of cell death. PGRN knockdown inhibited NSC34 cell proliferation in the presence of serum. This work supports the hypothesis that PGRN is neurotrophic and its actions are mediated by extracellular mechanisms.

## Methods

### *In situ *hybridization

ISH was conducted as described by [20] with slight modification. Paraffin sections were briefly de-paraffinized and rehydrated in a graded series of ethanol. Tissues were post-fixed in 4% paraformaldehyde (PFA) (pH7.4) and washed in 0.5× SSC buffer. Tissues were then permeabilized by incubation with proteinase K (3.5 μg/ml), post-fixed a second time with 4% PFA and then washed thoroughly in PBS and 0.5× SSC. Subsequently, the tissues were pre-hybridized for 3 hr and then hybridized with 1 ng/μl human-specific Dig-UTP-labelled 238 bp PGRN sense or antisense RNA probes in hybridization solution for ~18 h at 42°C. After washing, tissues were incubated with conjugated Dig antibody and reaction products were visualized with a brightfield Leica AS LMD microscope.

### Preparation of mouse spinal cord for Immunolabelling

Spinal cords were obtained from pregnant CD1 mice. Spinal cords were collected and fixed in 4% paraformaldehyde in PBS for 1 hour, cut into cervical, thoracic, lumbar and sacral sections, and then all were fixed overnight at the neuropathology department of the Montreal Neurological Institute. Each section was subsequently processed for paraffin sectioning. 4 μm slices were cut in the coronal plane and mounted onto positively charged slides (Fisher Scientific). Sections were dried in the oven overnight. then deparaffinized and rehydrated the next day (2 minute incubation in Citrisolv, 2 minute incubation in Citrisolv, 2 minute incubation in 100% ethanol, 2 minute incubation in 95% ethanol, 2 minute incubation in 75% ethanol, 2 minute incubation in 50% ethanol, rinsed in large volumes of water, washed in large volume PBS). Antigen retrieval was performed using a high pH buffer (Tris-EDTA with 0.05% Tween, pH 9.0), placed in a pressure cooker for 10 minutes. After cooling, the sections were placed in a 3% hydrogen peroxide solution to block endogenous peroxidase activity for 10 minutes. Sections were rinsed thoroughly in PBS and placed in blocking buffer (10% (w/v) horse serum, 5% bovine serum albumin (BSA), 0.3% Triton-X in PBS) for 1 hour. Sections were then processed according to the immunohistochemistry protocol outlined below.

### Primary spinal cord-DRG cultures

Dissociated primary motor neurons cultures were taken from embryonic day 13 (E13) mice, plated on either 25 mm or 14 mm coverslips (Electron Microscopy Sciences), and grown for 4 to 7 weeks after dissociation [64]. Cultures were fixed within the original plates using 4% PFA for 10 minutes, and incubated with permeabilization buffer (PBST with 0.2% Triton X-100) for 2 minutes, the cultures were post-fixed for 1 minute with 4%PFA, followed by incubation in blocking buffer (PBS with 5% (w/v) horse serum (Hyclone)) for one hour. Sections were then processed according to the immunohistochemistry protocol outlines below.

### Immunohistochemistry

All antibodies (primary and their corresponding secondary) used for immunohistochemistry are outlined in Table 1. After being incubated in appropriate blocking solution for the above mentioned periods of time, tissue sections and/or coverslips were then transferred to fresh blocking buffer containing the primary antibodies. Incubation with the primary antibody continued overnight at 4°C. Cultures were washed three times in PBS, and then incubated with the appropriate secondary antibodies in fresh blocking buffer for 45 minutes at room temperature. Cells were washed three times in PBS. Some cells were additionally counterstained using 300 nM DAPI in PBS for 5 minutes at room temperature in the dark. Cultures were washed three times with PBST, once with ddH_2_O, and then mounted onto slides using Immu-mount (Thermo Fisher), or fluorescent mounting medium (Dako). Fluorescence was visualized by confocal microscopy (LSM 510), using argon and HeNe1 lasers. Images were processed with Zen software. Identification of primary motor neurons within the heterogeneous culture was based upon SMI32 immunoreactivity and cell body size [64].

**Table 1:** List of primary and secondary antibodies used for immunocytochemistry

ANTIBODY	TITRE	SOURCE	SECONDARY
**PGRN**	1:500	R&D SYSTEMS	DONKEY-ANTI-SHEEP (ALEXA FLUOR 488 or 594
**TDP-43**	1:100	PROTEINTECH	DONKEY-ANTI-RABBIT (ALEXA FLUOR 488)
**CYTOCHROME C**	1:400	PHARMINGEN	DONKEY-ANTI-MOUSE (ALEXA FLUOR 488 OR 594)
**CALRETICULIN**	1:400	STRESSGEN	DONKEY-ANTI-RABBIT (ALEXA FLUOR 488 OR 594)
**GM130**	1:300	PHARMINGEN	DONKEY-ANTI-MOUSE (ALEXA FLUOR 488 OR 594)
**CHROMOGRANIN A**	1:100	RDI	DONKEY-ANTI-RABBIT (ALEXA FLUOR 488)
**SNAP-25**	1:1000	SIGMA	DONKEY-ANTI-RABBIT (ALEXA FLUOR 488)
**SYNAPTOPHYSIN**	1:200	SIGMA	DONKEY-ANTI-RABBIT (ALEXA FLUOR 488)
**CD11b**	1:100	BIOLEGENDS	NONE -(PRIMARY LABELED WITH ALEXA-FLUOR 488)
**GFAP**	1:400	PROTEINTECH	DONKEY-ANTI-RABBIT (ALEXA FLUOR 488)
**SMI32**	1:1000	STERNBERGER	DONKEY-ANTI-MOUSE (ALEXA FLUOR 488)

To control for nonspecific binding of the mouse PGRN antibody, antigen-competition was carried out by preadsorbing the antibody at 1:500 dilution with 300 ng/mL and 400 ng/mL recombinant mouse PGRN (Alexis Biochemicals). The mixture was incubated for 24 hr at 4°C and following centrifugation, the supernatant was collected and used as a primary antibody, as described above.

### Cloning and Transfection of pEGFP-N1-hPGRN vector

PCR primers were designed to include digestion sites for restriction enzymes. For pEGFP-N1-hPGRN, forward primer 5' C GAA TTC GAA TTC ACC ATG TGG ACC CTG GTG AGC 3' and reverse primer 5' GAC GTC GAC CCC AGC TGT CTC 3' were designed for EcoR1 and Sal1 digestion respectively. PCR was carried out under the following conditions: 94°C for 10 minutes, then 40 cycles of 94°C 1 min, 62°C 1 min, 72°C 1 min, followed by 10 minutes at 72°C. When cycle was finished, PCR block was kept at 4°C until samples were run on 1% agarose electrophoresis gel.

1 ug pEGFP-N1 plasmid and 1 ug single stranded *hpgrn *DNA was used in the restriction digest with EcoR1 and Sal1. Ligation mixtures were created as a 3:1 ratio, vector size to insert size respectively. 1 μl of T4 DNA Ligase (BioLabs) was added and tube was incubated at 20°C for 20 minutes. Competent cells (E. Coli, aliquots of 50 μl, Invitrogen) incubated on ice for 30 minutes, then heat shocked at 37°C for 2 minutes, followed by chilling on ice for 5 minutes. 950 μl LB broth (at room temperature) was added to the cells, which were subsequently incubated at 37°C for 1 hour. Cells were centrifuged at 12000 rpm for 30 seconds. 100 μl of the pellet (plus media) was spread onto a kanamycin coated LB plated Petri dishes and grown overnight in a 37°C incubator. Individual colonies were then amplified and confirmation of successful ligation was achieved through restriction enzyme digestion.

The NSC-34 cell line (generous gift from Dr. Neil Cashman) was maintained in DMEM with 10% fetal bovine serum unless otherwise stated [65]. NSC-34 cells were grown on 25 mm German glass coverslips in 6 well plates. At 70% confluency, cells were washed twice in serum-free OPTIMEM in preparation for transfection. Serum-free, antibiotic-free OPTIMEM and Lipofectamine were combined according to the manufacturer's instructions. For each coverslip, 4 ug of pEGFP-N1-hPGRN, or control empty vector pEGFP-N1, was added to the appropriate volume of OPTIMEM-lipofectamine solution and mixtures were incubated for 30 minutes prior to addition to well containing 1.5 ml of serum-free DMEM. Cells were incubated with the transfection mixture for 4 hours, after which the mixture was replaced with DMEM containing 10% FBS and gentamycin.

### NSC-34: Developing Stable Cell Lines

For stable transfections NSC-34 cells were transfected with human *PGRN *(*pcDNA-PGRN*) or empty vector (*pcDNA*) using Lipofectamine (Invitrogen) and selected with G418 for one month according to manufacturer's instructions.

#### RT-PCR for NSC-34/HPGRN cell line

To confirm the over-expression of human PGRN and to assess the expression of endogenous murine PGRN in NSC-34 cells, total RNA was isolated using Trizol reagent (Invitrogen) and 5 μg was used for cDNA synthesis using AMV reverse transcriptase (Invitrogen) according to manufacturer's instructions. Species-specific primer sets designed to detect human and mouse PGRN mRNA were 5'-GGACAGTACTGAAGACTCTG-3'/5'-GGATGGCAGCTTGTAATGTG-3' and 5'-GCTACAGACTTAAGGAACTC-3'/5'-GAAATGGCAGTTTGATACGG-3', respectively. Beta-actin mRNA was used as a loading control employing 5'-GAAGTGTGACGTGGACATCC-3' and 5'-GAAATGGCAGTTTGATACGG-3' primers. Polymerase chain reaction was completed using Platinum Taq (Invitrogen) with a denaturation of 2 min at 94°C; 35 cycles at 94°C, 30 sec; 55°C, 30 sec; 72°C, 30 sec; and a final extension of 5 min at 72°C.

#### Design and transfection of ShRNA -mPGRN into NSC-34 cells

The 29 nucleotide stretches within the coding region of murine PGRN cDNA unique in the genome were identified and an shRNA construct was designed by OriGene Technologies. NSC34 cells were cultured in DMEM with 10% fetal bovine serum unless otherwise stated [65]. For stable transfection, cells were seeded onto 6 well plates. When the cultures were 80% confluent, NSC-34 cells were transfected with shRNA-m *PGRN *(pRS/*shPGRN*) or control vector (pRS vector alone, Origene Technologies) using Fugene (Roche) and selected with puromycin (Invitrogen) for six weeks according to manufacturer's instructions.

#### RT-PCR for shPGRN cell line

To confirm the knockdown effect of murine PGRN in NSC-34 cells, total RNA was isolated using Trizol reagent (Invitrogen) and 5 μg was used for cDNA synthesis using AMV reverse transcriptase (Invitrogen) according to manufacturer's instructions. Species-specific primer set designed to detect mouse PGRN mRNA was 5'-GCTACAGACTTAAGGAACTC-3'/5'-GAAATGGCAGTTTGATACGG-3'. Beta-actin mRNA was used as a loading control employing 5'-GAAGTGTGACGTGGACATCC-3' and 5'-GAAATGGCAGTTTGATACGG-3' primers. Polymerase chain reaction was completed using Taq DNA polymerase (Invitrogen) with a denaturation of 2 min at 94°C; 35 cycles at 94°C, 30 sec; 55°C, 30 sec; 72°C, 30 sec; and a final extension of 5 min at 72°C.

#### Quantitative Real Time PCR (QRT-PCR)

QRT-PCR was performed as described elsewhere [41] using a Light Cycler FastStart DNA Master SYBR Green I kit (Roche). Melting curve analysis confirmed the presence of a single product for every PCR primer used, and genomic contamination was excluded by amplification of a control sample without reverse transcription. Quantitation was calculated using the formula:

where RER is relative expression ratio, *E *is the efficiency of the PCR reaction, ΔCP is the difference in crossing points and ref is the corresponding value for a reference gene (Actin) [77]. PCR primers (as described for conventional RT-PCR) were synthesized by Invitrogen. Each RT-PCR experiment was performed using 3 independent RNA extracts in duplicate.

### Western blot analysis

NSC34 cells were lysed and proteins were solubilized in RIPA buffer (Sigma) containing Complete Protease Inhibitor Cocktail (Roche Applied Science). Lysates were incubated on ice for 5 min, briefly sonicated and centrifuged at 13,000 rpm for 15 min at 4°C. Supernatants were mixed with equal amount of in 2× sample buffer boiled for 5 min, and resolved on a 10% SDS-PAGE gel. Proteins were transferred onto a nitrocellulose membrane and blocked over-night with membrane blocking agent (GE Healthcare) at 4°C. The blots were incubated in PBST with 1:250 anti-human PGRN polyclonal antibody (rabbit anti-human PGRN generated in our laboratory) for 1 hour followed by extensive washing. After incubating with an anti-rabbit or -mouse IgG-horseradish peroxidase (HRP)-conjugated secondary antibody (diluted 1:4,000) at room temperature for 1 hour and blots were visualized using enhanced chemiluminescence (GE Healthcare) according to the manufacturer's instructions. The same blot was stained with mouse monoclonal β-actin antibody (AC-40; Sigma) at a dilution of 1:1000 as a control for total protein loading.

### Survival Assays

Serum deprivation assays were carried out in 6-well plates using 200,000 cells/well and cultured in 4 ml of RPMI (with glutamine) for 3, 6, 9, 12 and 15 days without the addition or exchange of fresh medium. For each time point the average cell number was determined over 6 visual fields per well at 10× magnification using an Olympus phase-contrast microscope. For long term cultures NSC-34 cells were plated at a density of 200,000/well in 6-well plates and maintained in serum free RPMI medium. Fresh serum-free medium was provided every 10 days and 10× magnification photos taken at intervals for 2 months using an Olympus phase-contrast microscope.

For stimulation with exogenous PGRN, NSC-34 cells were plated in 96 well plates at 6000 cells per well. The following day the cultures were treated with or without PGRN at 100 ng/ml under serum-free conditions in RPMI for 5 days. The PGRN was prepared by transient transfection in COS7 cells as previously described, and purified to homogeneity by reversed-phase high pressure liquid chromatography [78]. The purity and identity of the protein was confirmed by SDS-gel electrophoresis followed by silver staining and Western blotting. Cell viability was assessed using the AlamarBlue (Biosource, Camarillo, CA) colorimetric assay. The AlamarBlue assay allows quantitative analysis of cell viability due to metabolic activity that results in a chemical reduction of AlamarBlue from the oxidized (blue) form to the reduced (pink) form. Plates were read in an ELISA plate reader at 2 different wavelengths, 570 nm and 630 nm 24 hrs after addition of AlamarBlue.

### Apoptosis TUNEL assay

NSC-34 cells were plated on photo-etched German glass coverslips (Electron Microscopy sciences) in 6-well plates at 200,000/well and cultured in 4 ml of RPMI (with glutamine) for six days. At time of fixation, cells were washed twice in PBS, and then fixed using 4% PFA/PBS for 20 min. After being rinsed three times in PBST, cells were incubated in permeabilization buffer (0.2% Triton X-100 in PBST) for 20 min. Cells were subsequently post-fixed for 10 min with 4% PFA/PBS. After being washed extensively with PBST, they were stored at 4°C in sterile PBS. At time of processing, cells were rinsed once with PBS, followed by overlay with reaction solution from the Fluorescein *In Situ *Death Detection Kit (Roche Applied Science), as directed by manufacturer's instructions. Cells were incubated at 37°C for 1 hr, and then rinsed twice with PBST at room temperature in the dark. After rinsing three times in PBST, cells were counterstained with 300 nM 4', 6-diamino-2-phenylindole (DAPI) for 5 min in the dark. Cells were then rinsed twice with PBST, once with ddH_2_O and then mounted onto slides using Immu-mount (Thermo Fisher). Fluorescence was visualized with an Axioskop2 microscope equipped with appropriate filters and total cells (DAPI) versus apoptotic cells (FITC) were counted manually by visual inspection.

### Chemiluminescent BrdU proliferation assay

NSC-34 cells were plated in 96-well plates at 6000/well and cultured in RPMI (with glutamine) for 5 days. Eighteen hours prior to fixation/processing, BrdU labeling solution (Roche Applied Sciences) was added to each well at a concentration of 10 μM. At the time of fixation, the excess unincorporated BrdU labeling medium was removed and cells were dried and then fixed using FixDenat solution (Roche Applied Sciences) for 30 min. FixDenat solution was decanted and the cells were incubated with anti-BrdU-POD (1:100) for 120 min. Cells were rinsed three times with washing solution for five min each. Cells were then placed in substrate solution for 3 min at room temperature on a sample shaker. The light emission from the samples was measured using microplate a luminometer (LMAX II-384, Molecular Devices) with photomultiplier technology and expressed as relative light units per second (rlu/s).

### AlamarBlue Cell Proliferation Assay

NSC 34 cells were plated in 96 well plates and cultured in DMEM with 10% fetal bovine serum. For rescue experiments the cells were cultured with or without 200 ng/ml of human PGRN at day 1. Cell number was assessed using the AlamarBlue (Biosource, Camarillo, CA) colorimetric assay from day0 to day7. Plates were read in an ELISA plate reader at 2 different wavelengths, 570 nm and 630 nm 24 hrs after addition of AlamarBlue. Cell based Standard curve was prepared using 2000, 6000, 10000, 20000 and 50000 cells per well and corresponding absorbance values. Cell numbers of unknowns were calculated using Standard curve.

## Abbreviations

ALS: Amyotrophic Lateral Sclerosis; DRG: Dorsal root ganglia; FTLD: frontotemporal lobar degeneration; FTLD-U: frontotemporal lobar degeneration with ubiquitin positive inclusions; GFP: Green fluorescent protein; GRN: granulin domain; ISH: In situ hybridization; PGRN: progranulin; SOD: Superoxide dismutase; TDP-43: TAR-DNA binding protein 43; TGN: Trans-Golgi network.

## Authors' contributions

David Baranowski and Cara Ryan undertook the NSC-34 cell survival and TUNEL analyses, and localization of PGRN in spinal cord sections and NSC-34 cells. Cara Ryan performed the subcellular localization of PGRN in primary spinal cord motor neurons by confocal microscopy, as well as the cloning, preparation and transfection of the hPGRN-eGFP-N1 construct. Babykumari Chitramuthu performed the NSC-34 cell survival assay in response to exogenous PGRN and BrdU analysis, established and validated the shPGRN-NSC34 stable cell line by RT-PCR, qRT-PCR and western blot analysis, and carried out cell proliferation, rescue and TUNEL assays on PGRN deficient NSC34 cells. Zhi Li prepared and characterised the recombinant human PGRN. Suneil Malik established the NSC-34/PGRN cells. Ming Cao performed the *in situ *hybridizations. Sandra Minotti and Heather D. Durham provided the primary spinal cord-DRG cultures and advised on several aspects of the work. Denis G. Kay assisted with interpretation of *in situ *hybridization and contributed to the design of the study. Christopher A. Shaw provided the spinal cord sections and contributed to the design of the study. Andrew Bateman undertook the long-term proliferation experiments with NSC-34/PGRN. Hugh P.J. Bennett and Andrew Bateman designed the study, coordinated its execution and finalized the manuscript. All co-authors read and approved the final manuscript.
